# Effects of short-term PM_2.5_ exposure on blood lipids among 197,957 people in eastern China

**DOI:** 10.1038/s41598-023-31513-y

**Published:** 2023-03-18

**Authors:** Qiao Liu, Zhan Wang, Junjie Lu, Zhongqi Li, Leonardo Martinez, Bilin Tao, Chunlai Wang, Limei Zhu, Wei Lu, Baoli Zhu, Xiaohua Pei, Xuhua Mao

**Affiliations:** 1 Department of Chronic Communicable Disease, Center for Disease Control and Prevention of Jiangsu Province, Nanjing, Jiangsu Province People’s Republic of China; 2 Department of Epidemiology, Center for Global Health, School of Public Health, Nanjing Medical University, Nanjing, People’s Republic of China; 3 Department of Critical Care Medicine, Affiliated Yixing People’s Hospital, Jiangsu University, Wuxi, People’s Republic of China; 4 Department of Epidemiology, School of Public Health, Boston University, Boston, MA USA; 5 Department of Physical Examination Center, Affiliated Yixing People’s Hospital, Jiangsu University, Wuxi, People’s Republic of China; 6 Divison of Geriatric Nephrology, The First Affiliated Hospital of Nanjing Medical University, Nanjing, Jiangsu Province People’s Republic of China; 7 Department of Clinical Laboratory, Affiliated Yixing People’s Hospital, Jiangsu University, Wuxi, Jiangsu Province People’s Republic of China

**Keywords:** Environmental sciences, Environmental social sciences, Cardiovascular diseases

## Abstract

Globally, air pollution is amongst the most significant causes of premature death. Nevertheless, studies on the relationship between fine particulate matter (PM_2.5_) exposure and blood lipids have typically not been population-based. In a large, community-based sample of residents in Yixing city, we assessed the relationship between short-term outdoor PM_2.5_ exposure and blood lipid concentrations. Participants who attended the physical examination were enrolled from Yixing People’s hospital from 2015 to 2020. We collected general characteristics of participants, including gender and age, as well as test results of indicators of blood lipids. Data on daily meteorological factors were collected from the National Meteorological Data Sharing Center (http://data.cma.cn/) and air pollutant concentrations were collected from the China Air Quality Online Monitoring and Analysis Platform (https://www.aqistudy.cn/) during this period. We applied generalized additive models to estimate short-term effects of ambient PM_2.5_ exposure on each measured blood lipid-related indicators and converted these indicators into dichotomous variables (non- hyperlipidemia and hyperlipidemia) to calculate risks of hyperlipidemia associated with PM_2.5_ exposure. A total of 197,957 participants were included in the analysis with mean age 47.90 years (± SD, 14.28). The increase in PM_2.5_ was significantly associated with hyperlipidemia (odds ratio (OR) 1.003, 95% CI 1.001–1.004), and it was still significant in subgroups of males and age < 60 years. For every 10 μg/m^3^ increase in PM_2.5_, triglyceride levels decreased by 0.5447% (95% CI − 0.7873, − 0.3015), the low-density lipoprotein cholesterol concentration increased by 0.0127 mmol/L (95% CI 0.0099, 0.0156), the total cholesterol concentration increased by 0.0095 mmol/L (95% CI 0.0053, 0.0136), and no significant association was observed between PM_2.5_ and the high-density lipoprotein cholesterol concentration. After excluding people with abnormal blood lipid concentrations, the associations remained significant except for the high-density lipoprotein cholesterol concentration. PM_2.5_ was positively correlated with low-density lipoprotein cholesterol and total cholesterol, and negatively correlated with triglyceride, indicating PM_2.5_ can potentially affect health through blood lipid levels.

## Introduction

Exposure to fine particulate matter (PM_2.5_) has been linked to a substantial disease burden globally^1^. Although some research has shown that short-term exposure to PM_2.5_ is positively correlated with mortality from respiratory diseases as well as an increased risk of cardiovascular disease^2,3^, the mechanisms and other impacts of PM_2.5_ exposure on health is still unclear.

Previous studies have reported that PM_2.5_ exposure may increase the incidence of non-alcoholic fatty liver disease^4^. A previous study among senior citizens found that individuals exposed to long-term PM_2.5_ exposure had an increased incidence of dementia^5^. Other studies found long-term PM_2.5_ exposure was associated with increased serum triglyceride and decreased high-density lipoprotein cholesterol concentration in elderly males^6^. Other research in children and adolescents suggests that long-term PM_2.5_ exposure was positively associated with the total cholesterol concentration and risk of hypercholesterolemia^7^. Whether PM_2.5_ exposure and blood lipids are epidemiologically related is debated and few large studies have investigated this relationship at the population-level.

Although there have been several studies on PM_2.5_ exposure and blood lipids, most of these studies are based on long-term PM_2.5_ exposure, and few studies have explored the association between short-term PM_2.5_ exposure and blood lipids. To further the understanding of the relationship between short-term PM_2.5_ exposure and blood lipids, we collected test results of blood lipid-related indicators through routine physical examinations from a community-based sample of 197,957 residents in Yixing city. We also assessed for a range of environmental factors during the same period.

## Methods

### Study population

This cross-sectional study was performed in Yixing city, located in eastern China, with a population of approximately 1.3 million. The study population was not selected based on disease status; participants who attended a routine physical examination at Yixing People’s Hospital from 2015 to 2020 were eligible and enrolled in the study. No subjects repeatedly took part in the study. Inclusion criteria: (1) participants who were tested for lipid-related indicators (2) participants were local residents Exclusion criteria: (1) participants who took lipid-lowering drugs (2) participants who were workers exposed to dust. We collected participant characteristics, blood lipid-related indicators, including total cholesterol, triglyceride, low-density lipoprotein cholesterol, and high-density lipoprotein cholesterol. Blood samples were obtained from individuals after at least 8 hours overnight fasting. High-density lipoprotein cholesterol and low-density lipoprotein cholesterol was analyzed by the direct assay method. Total cholesterol by cholesterol oxidase method and triglyceride by enzymatic method, using CobasC501, (Roche Diagnostics GmbH, Switzerland).

### Binary and continuous outcomes

According to Chinese guidelines for the prevention and treatment of dyslipidemia in adults^8^, the normal range of these indicators are: (1) total cholesterol < 6.2 mmol/L; (2) triglyceride < 2.3 mmol/L; (3) low-density lipoprotein cholesterol < 4.1 mmol/L; and (4) high-density lipoprotein cholesterol > 1.0 mmol/L. We calculated the number of participants with normal blood lipid-related indicators separately. Participants with abnormalities in either indicator were defined as having hyperlipidemia.

### Data on meteorological factors and air pollutants

The exposure data were obtained from a fixed monitoring station ((120.35′E, 31.62′N)) for the city. The quality control methods of the monitoring stations include climate limit value check, station extreme value check, time consistency check, space consistency check and manual check. We collected daily average meteorological factors, including atmospheric pressure (hPa), temperature (^o^C), wind speed (m/s), and relative humidity (%) during January 8, 2015 and December 31, 2020 from the National Meteorological Data Sharing Center (http://data.cma.cn/). Data on daily average air pollutant concentrations, including PM_2.5_, PM_10_ (particles of less than 10 μm diameter), sulphur dioxide (SO_2_), nitrogen dioxide (NO_2_), ozone (O_3_), and carbon monoxide (CO) were collected from the China Air Quality Online Monitoring and Analysis Platform (https://www.aqistudy.cn/). Except that the unit of CO concentration was mg/m^3^, the unit of other pollutants was μg/m^3^.

### Statistical analysis

A generalized additive model (GAM) was applied to explore the relationship between short-term ambient PM_2.5_ exposure and blood lipid-related indicators similar to prior studies^9,10^. GAMs are useful for evaluating the impact of air pollution on human health^11^. Among the four indicators, triglyceride were not normally distributed. Total cholesterol, low-density lipoprotein cholesterol and high-density lipoprotein cholesterol were all normally distributed. We performed natural log conversions of triglyceride to achieve a approximate normal distribution. To account for potential confounders, adjusted covariates in the GAM model included day of the week, time, sex, age, and meteorological factors. To address multiple collinearities, Spearman rank correlation coefficients between environmental factors were calculated; the model only included variables with $\left|\mathrm{r}\right|$<0.7^12^. We applied a thin plate spline function in order to control for nonlinear effects of meteorological factors^13^. Minimum Akaike information criterion (AIC) values corresponded to the optimal degree of freedom^10^. Considering lag effects of PM_2.5_ on blood lipids, we calculated 2- to 8-day moving averages (lag 0–1 day to lag 0–7 days) of the daily average concentration of PM_2.5_ to capture cumulative lag effects. For example, if a person attended the physical examination on January 9, we collected the daily average concentration of PM_2.5_ from January 2 to January 9, and then calculated the average concentration from January 8 to January 9 as the 2-day moving average. The n-day moving average concentration was applied to estimate personal short-term PM_2.5_ exposure level. Minimizing the AIC value was applied to identify the optimal lag time^14–16^. We expressed the effects as the estimated changes in blood lipid-related indicators and their 95% confidence intervals (CIs) for a 10 μg/m^3^ increase in ambient PM_2.5_ concentration^15^. We also converted lipid-related indicators into dichotomous variables (normal and abnormal) to calculate risks of hyperlipidemia associated with PM_2.5_ exposure, and expressed them as the odds ratio (OR) as well as their 95% CIs for 10 μg/m^3^ rise in outdoor PM_2.5_ concentration. In addition, we analyzed the relationship between other air pollutions (including PM_10_, SO_2_, NO_2_, O_3_, and CO) and blood lipids using similar approaches.

We performed two sensitivity analyses to examine the robustness of the associations between PM_2.5_ and blood lipid-related indicators. First, we constructed single- and multi-pollutant models for PM_2.5_, respectively. Second, individuals with abnormal indicators were excluded to estimate the effects of PM_2.5_ among the population with normal indicators. A subgroup analysis was also performed to explore if the effect was modified by sex or age. The heterogeneity effects between subgroups were evaluated using the formula: $\left|{\beta }_{1}-{\beta }_{2}\right|/\sqrt{{{SE}_{1}}^{2}+{{SE}_{2}}^{2}}$, where ${\beta }_{1}$ and ${\beta }_{2}$ are the estimated effects, and ${SE}_{1}$ and ${SE}_{2}$ are their standard errors, respectively. When the value was larger than 1.96, the difference was considered statistically significant^15^.

All analyses were performed with the “mgcv” and “ggplot2” packages in R software version 4.1.2 (https://www.r-project.org/). The significance level was set at 0.05.

### Ethics statement

This study was approved by ethics committee of Yixing people’s hospital.

## Results

### Characteristics of study participants

**Figure 1:**
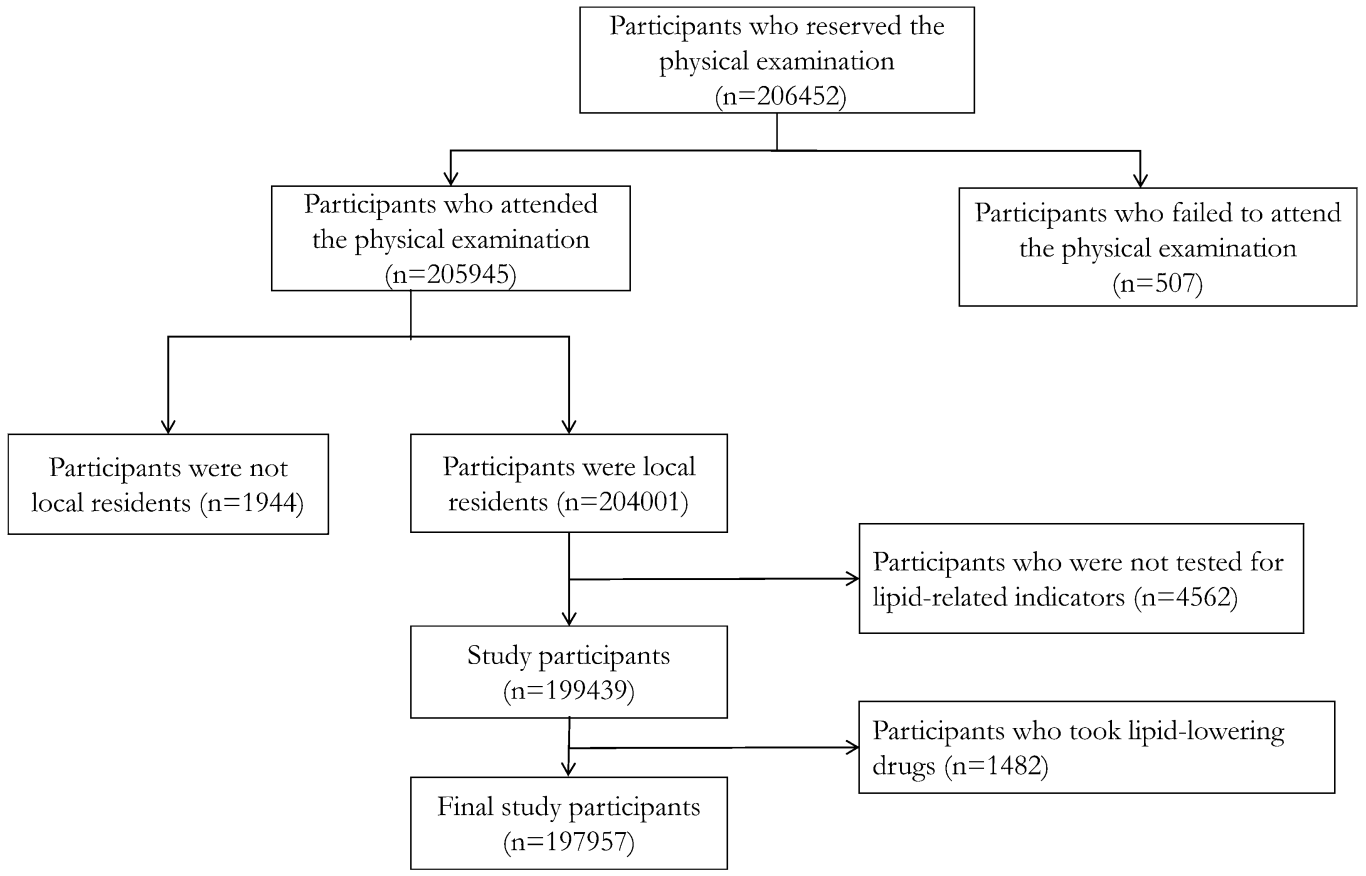
Flowchart of participants enrolled in the study from eastern China.

Of 206,452 participants eligible for the study, 205,945 attended a physical examination. In total, 7988 participants were excluded for various reasons; 1944 (0.90%) participants were excluded because they were not local residents while 4562 participants were not tested for lipid-related indicators. Lastly, 1482 participants were taking lipid-lowering drugs during the study period and were also excluded (Fig. 1). After exclusions, a total of 197,957 people were included in the analysis. Mean age was 47.90 years (± SD, 14.28) and 55.61% of participants were male. The mean values of total cholesterol, low-density lipoprotein cholesterol, and high-density lipoprotein cholesterol was 4.94 mmol/L (± 0.94), 2.73 mmol/L (± 0.67), and 1.31 mmol/L (± 0.31), respectively. The median triglyceride values were 1.35 mmol/L ((interquartile range [IQR], 0.92, 2.04) (Table 1). The number of participants with normal levels was 133,080 for triglyceride, 132,643 for low-density lipoprotein cholesterol, 132,308 for high-density lipoprotein cholesterol and 133,172 for total cholesterol.

**Table 1:** Characteristics of the study population.

Variables	N (%)	Median (IQR)	Mean (± SD)
Sex			
Male	110,090 (55.61)		
Female	87,867 (44.39)		
Age		48.00 (37.00–57.00)	47.90 ± 14.28
< 60 years	156,195 (78.90)	44.00 (34.00–51.00)	42.46 ± 10.20
≥ 60 years	41,762 (21.10)	66.00 (63.00–72.00)	68.27 ± 7.19
Hyperlipidemia	64,448 (32.56)		
Low-density lipoprotein cholesterol (mmol/L)		2.66 (2.22–3.13)	2.73 ± 0.67
High-density lipoprotein cholesterol (mmol/L)		1.27 (1.08–1.49)	1.31 ± 0.31
Total cholesterol (mmol/L)		4.87 (4.30–5.50)	4.94 ± 0.94
Triglyceride (mmol/L)		1.35 (0.92–2.04)	1.72 ± 1.44
Meteorological factors			
Temperature (°C)		18.10 (9.60–24.70)	17.47 (± 8.97)
Atmospheric pressure (hPa)		1016.30 (1008.00–1023.40)	1016.16 (± 9.35)
Wind speed (m/s)		2.10 (1.50–2.60)	2.14 (± 0.83)
Relative humidity (%)		74.00 (64.00–83.00)	73.40 (± 13.37)
Air pollutants			
PM_2.5_ (μg/m^3^)		38.00 (26.00–57.00)	45.56 (± 28.45)
PM_10_ (μg/m^3^)		67.00 (48.00–95.00)	76.64 (± 41.12)
SO_2_ (μg/m^3^)		11.00 (8.00–18.00)	13.86 (± 8.81)
NO_2_ (μg/m^3^)		38.00 (28.00–51.00)	41.76 (± 17.82)
CO (mg/m^3^)		0.90 (0.70–1.10)	0.96 (± 0.33)
O_3_ (μg/m^3^)		95.00 (63.00–140.00)	103.50 (± 51.59)

### Characteristics of meteorological factors and air pollutants

The median daily average meteorological factors and air pollutant concentrations was 18.10 °C for temperature, 1016.30 hPa for atmospheric pressure, 2.10 m/s for wind speed, 74% for relative humidity, 38.00 μg/m^3^ for PM_2.5_, 67.00 μg/m^3^ for PM_10_, 11.00 μg/m^3^ for SO_2_, 38.00 μg/m^3^ for NO_2_, 0.90 mg/m^3^ for CO, and 95.00 μg/m^3^ for O_3_ (Table 1). PM_2.5_ was positively correlated with atmospheric pressure, PM_10_, SO_2_, NO_2_, and CO, and negatively correlated with temperature and wind speed (*P* < 0.05). Because Spearman rank correlation coefficients between PM_2.5_ and PM_10_ and CO were larger than 0.7, the above two air pollutants were excluded from the final model. The absolute value of the correlation coefficient between temperature and atmospheric pressure was larger than 0.7, atmospheric pressure was removed from the model (Supplementary Table 1). The df of meteorological factors in the analyze of effects of PM_2.5_ on blood lipids were shown in Supplementary Table 2.

### PM_2.5_ and blood lipids in entire population

**Figure 2:**
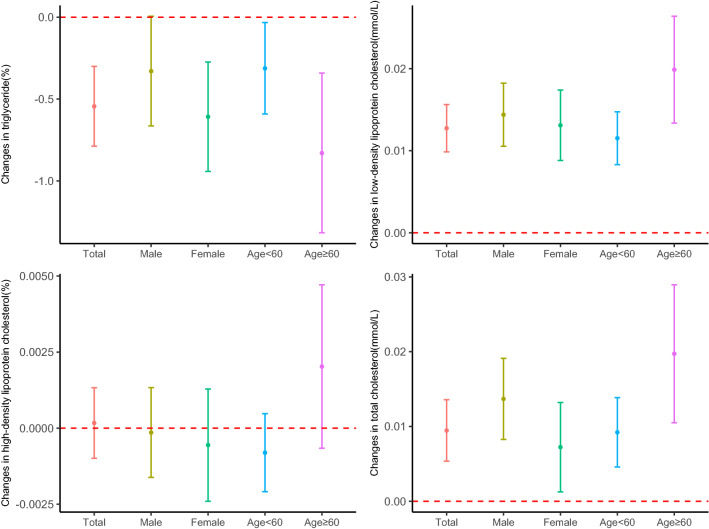
Estimated changes (95% confidence intervals) in the blood lipids for every 10 μg/m^3^ increase in PM_2.5_ among the entire population. We applied lag 0–6 days for triglyceride, lag 0–7 days for low-density lipoprotein cholesterol, lag 0–5 days for high-density lipoprotein cholesterol, and lag 0–7 days for total cholesterol.

We applied lag 0–6 days, 0–7 days, 0–5 days and 0–7 days for triglyceride, low-density lipoprotein cholesterol, high-density lipoprotein cholesterol and total cholesterol in the entire population respectively. For a 10 μg/m^3^ increase in PM_2.5_, the triglyceride decreased by 0.5447% (95% CI − 0.7873, − 0.3015), the low-density lipoprotein cholesterol concentration increased by 0.0127 mmol/L (95% CI 0.0099, 0.0156) and the total cholesterol concentration increased by 0.0095 mmol/L (95% CI 0.0053, 0.0136) and no significant association was observed between PM_2.5_ and the high-density lipoprotein cholesterol concentration(Table 2). The associations remained significant of low-density lipoprotein cholesterol and total cholesterol by the subgroups of males, females, age < 60 years and age ≥ 60 years. Of triglyceride, the associations remained significant in the subgroups of females, age < 60 years and age ≥ 60 years, and the effect of short-term PM_2.5_ exposure on the low-density lipoprotein cholesterol concentration and total cholesterol concentration could be modified by age, the effects was stronger for the subgroup of age ≥ 60 years (Fig. 2, Supplementary Table 3).

**Table 2:** Estimated changes in the blood lipids for every 10 μg/m^3^ increase in PM_2.5_.

Indicators	Complete study population^a^		Persons with normal blood lipid levels^a^	
	Single-pollutant model	Multi-pollutant model^b^	Single-pollutant model	Multi-pollutant model^b^
Triglyceride (%)	− 0.5447 (− 0.7873, − 0.3015)	0.3081 (− 0.0495, 0.6669)	− 0.5184 (− 0.7235, − 0.3128)	0.2006 (− 0.1024, 0.5045)
Low-density lipoprotein cholesterol (mmol/L)	0.0127 (0.0099, 0.0156)	0.0194 (0.0152, 0.0237)	0.0096 (0.0068, 0.0124)	0.0134 (0.0093, 0.0175)
High-density lipoprotein cholesterol (mmol/L)	0.0002 (− 0.0010, 0.0013)	− 0.0022 (− 0.0039, − 0.0005)	− 0.0002 (− 0.0014, 0.0011)	− 0.0017 (− 0.0036, 0.0001)
Total cholesterol (mmol/L)	0.0095 (0.0053, 0.0136)	0.0284 (0.0224, 0.0345)	0.0057 (0.0019, 0.0095)	0.0209 (0.0153, 0.0265)

^a^Persons with normal blood lipid levels were people with total cholesterol < 6.2 mmol/L, triglyceride < 2.3 mmol/L, low-density lipoprotein cholesterol < 4.1 mmol/L and high-density lipoprotein cholesterol > 1.0 mmol/L. We applied lag 0–6 days for triglyceride, lag 0–7 days for low-density lipoprotein cholesterol, lag 0–5 days for high-density lipoprotein cholesterol, and lag 0–7 days for total cholesterol.

^b^Adjusted for SO_2_, NO_2_ and O_3_.

### PM_2.5_ and blood lipids in persons with normal blood lipid levels

**Figure 3:**
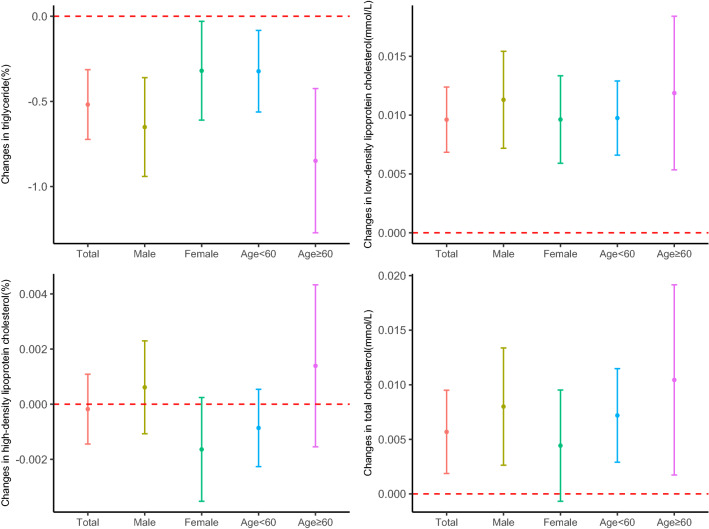
Estimated changes (95% confidence intervals) in the blood lipids for every 10 μg/m^3^ increase in PM_2.5_ among persons with normal blood lipid levels. We applied lag 0–6 days for triglyceride, lag 0–7 days for low-density lipoprotein cholesterol, lag 0–5 days for high-density lipoprotein cholesterol, and lag 0–7 days for total cholesterol.

In persons with normal test results, for a 10 μg/m^3^ increase in PM_2.5_, the triglyceride decreased by 0.5184% (95% CI − 0.7235, − 0.3128), the low-density lipoprotein cholesterol concentration increased by 0.0096 mmol/L (95% CI 0.0068, 0.0124) and the total cholesterol concentration increased by 0.0057 mmol/L (95% CI 0.0019, 0.0095). No significant association was observed between PM_2.5_ and the high-density lipoprotein cholesterol concentration (Table 2). The associations remained significant of low-density lipoprotein cholesterol and triglyceride in the subgroups of males, females, age < 60 years and age ≥ 60 years and remained significant of the total cholesterol concentration in the subgroups of males, age < 60 years and age < 60 years (Fig. 3). After excluding participants with abnormal test results, short-term PM_2.5_ exposure and its effect on triglyceride could be modified by age, the effects were stronger for the subgroup of age ≥ 60 years (Supplementary Table 3).

### The effects of PM_2.5_ on the blood lipid at different lag days

The associations between PM_2.5_ and triglyceride, low-density lipoprotein cholesterol concentration and total cholesterol concentration were robust at different lag days. And the effects of PM_2.5_ exposure on triglyceride, low-density lipoprotein cholesterol concentration, and total cholesterol concentration were strongest at lag 0–4 days, lag 0–7 days, lag 0–4 days and lag 0-7 days. However, no significant association was observed between PM_2.5_ and the high-density lipoprotein cholesterol concentration at different lag days. (Table 3).

**Table 3:** Estimated changes in the blood lipids for every 10 μg/m^3^ increase in PM_2.5_ at different lag days.

Indicators and lag days	Entire population	Persons with normal blood lipid levels^a^
Triglyceride (%)		
0–1 days	− 0.3718 (− 0.5286, − 0.2147)	− 0.2735 (− 0.4070, − 0.1399)
0–2 days	− 0.5087 (− 0.6875, − 0.3296)	− 0.4713 (− 0.6235, − 0,.3188)
0–3 days	− 0.5394 (− 0.7371, − 0.3414)	− 0.5895 (− 0.7580, − 0.,4206)
0–4 days	− 0.5895 (− 0.8039, − 0.3745)	− 0.6790 (− 0.8621, − 0.4956)
0–5 days	− 0.5102 (− 0.7400, − 0.2798)	− 0.5734 (− 0.7701, − 0.3764)
0–6 days	− 0.5447 (− 0.7873, − 0.3015)	− 0.5184 (− 0.7235, − 0.3128)
0–7 days	− 0.5376 (− 0.7873, − 0.2874)	− 0.5297 (− 0.7341, − 0.3185)
Low-density lipoprotein cholesterol (mmol/L)		
0–1 days	0.0055 (0.0037, 0.0073)	0.0020 (0.0002, 0.0037)
0–2 days	0.0051 (0.0030, 0.0071)	0.0011 (-0.0009, 0.0031)
0–3 days	0.0064 (0.0041, 0.0086)	0.0022 (0.0000, 0.0044)
0–4 days	0.0074 (0.0049, 0.0099)	0.0038 (0.0014, 0.0062)
0–5 days	0.0082 (0.0056, 0.0108)	0.0050 (0.0024, 0.0076)
0–6 days	0.0104 (0.0076, 0.0132)	0.0071 (0.0044, 0.0099)
0–7 days	0.0127 (0.0099, 0.0156)	0.0096 (0.0068, 0.0124)
High-density lipoprotein cholesterol (mmol/L)		
0–1 days	0.0005 (− 0.0003, 0.0013)	0.0002 (− 0.0006, 0.0011)
0–2 days	0.0004 (− 0.0005, 0.0013)	0.0001 (− 0.0009, 0.0011)
0–3 days	0.0004 (− 0.0006, 0.0014)	0.0001 (− 0.0009, 0.0012)
0–4 days	0.0008 (− 0.0003, 0.0019)	0.0005 (− 0.0007, 0.0017)
0–5 days	0.0002 (− 0.0010, 0.0013)	− 0.0002 (− 0.0014, 0.0011)
0–6 days	0.0000 (− 0.0013, 0.0012)	− 0.0005 (− 0.0019, 0.0008)
0–7 days	0.0005 (− 0.0008, 0.0018)	− 0.0002 (− 0.0016, 0.0011)
Total cholesterol (mmol/L)		
0–1 days	0.0067 (0.0041, 0.0092)	0.0033 (0.0010, 0.0056)
0–2 days	0.0073 (0.0044, 0.0102)	0.0031 (0.0005, 0.0057)
0–3 days	0.0076 (0.0044, 0.0108)	0.0032 (0.0002, 0.0061)
0–4 days	0.0080 (0.0046, 0.0114)	0.0039 (0.0007, 0.0070)
0–5 days	0.0083 (0.0045, 0.0120)	0.0051 (0.0018, 0.0085)
0–6 days	0.0086 (0.0047, 0.0126)	0.0054 (0.0017, 0.0091)
0–7 days	0.0095 (0.0053, 0.0126)	0.0057 (0.0019, 0.0095)

Adjusted for time, day of the week, sex, age, temperature, wind speed and relative humidity.

^a^Persons with normal blood lipid levels were people with total cholesterol < 6.2 mmol/L, triglyceride < 2.3 mmol/L, low-density lipoprotein cholesterol < 4.1 mmol/L and high-density lipoprotein cholesterol > 1.0 mmol/L.

### The effects of PM_2.5_ on the blood lipids in multi-pollutant models

For a 10 μg/m^3^ increase in PM_2.5_, the low-density lipoprotein cholesterol concentration increased by 0.0194 mmol/L (95% CI 0.0152, 0.0237), the high-density lipoprotein cholesterol concentration decreased by 0.0022 mmol/L (95% CI − 0.0039, − 0.0005) and the total cholesterol concentration increased by 0.0284 mmol/L (95% CI 0.0224, 0.0345). No significant association was observed between PM_2.5_ and the triglyceride. In persons with normal test results, for a 10 μg/m^3^ increase in PM_2.5_, the low-density lipoprotein cholesterol concentration increased by 0.0134 mmol/L (95% CI 0.0093, 0.0175) and the total cholesterol concentration increased by 0.0209 mmol/L (95% CI 0.0153, 0.0265). No significant association was observed between PM_2.5_ and the triglyceride and high-density lipoprotein cholesterol concentration (Table 2).

### The effects of PM_2.5_ on hyperlipidemia

We converted lipid-related indicators into binary variables (non- hyperlipidemia and hyperlipidemia) to calculate risks of hyperlipidemia associated with PM_2.5_ exposure. As a result, when PM_2.5_ increased 10 μg/m^3^, the OR (95% CIs) was 1.003 (95% CI 1.001, 1.004), and it was still significant in the subgroups of males and age < 60 years (Supplementary Table 4). We also converted lipid-related indicators into binary variables (normal and abnormal) to calculate the OR and 95% CI of blood lipids for every 10 μg/m^3^ increase in PM_2.5_. We applied lag 0–6 days for triglyceride, lag 0–3 days for low-density lipoprotein cholesterol, lag 0–5 days for high-density lipoprotein cholesterol and lag 0–7 days for total cholesterol. When PM_2.5_ increased 10 μg/m^3^, the OR (95% CIs) for triglyceride, low-density lipoprotein cholesterol, high-density lipoprotein cholesterol and total cholesterol was 0.998 (95% CI 0.996, 0.999), 1.001 (95% CI 1.000, 1.001), 1.002 (95% CI 1.001, 1.003) and 1.003 (95% CI 1.001, 1.004) (Supplementary Table 5).

The effects of other air pollutions (including PM_10_, SO_2_, NO_2_, O_3_, and CO) on the blood lipids are shown in the Supplementary Appendix (Supplementary Tables 6–15).

## Discussion

In this study, we found that PM_2.5_ was positively correlated with low-density lipoprotein cholesterol concentration and total cholesterol concentration, while being negatively correlated with triglyceride. Findings from our study provide evidence of the potential harmful effects of PM_2.5_ exposure on blood lipids. To our knowledge, this is the largest population-based study to explore the association between short-term PM_2.5_ exposure and blood lipids, and will provide new empirical for the effect of short-term air pollutant exposure on health.

Previous studies have been heterogeneous with some showing similar results^17,18^ while others harmful. Study design and differential exposure may partially explain these differences. For example, long-term exposure to PM_2.5_ was positively associated with triglyceride concentration in a study in Perth^6^. Distinct durations of exposure may partially explain this inconsistency and our analysis specifically evaluated short-term effect of PM_2.5_ exposure while many studies concentrated on longer-term effect of PM_2.5_^6^. Previous studies in rural areas have demonstrated that short-term PM_2.5_ exposure was positively associated with triglyceride concentration^19^ and negatively associated with total cholesterol concentration^20^, inconsistent with our results. Differences in lifestyle and air quality between rural and urban areas may influence outcomes^17,18,21^. For example, Omega-3 fatty acids may attenuate cardiovascular effects of short-term exposure to ambient air pollution^22^. In our study, PM_2.5_ was negatively correlated with triglyceride in the single pollutant model, and positively correlated with triglyceride in the multi-pollutant model, regardless of the overall population or the population with normal blood lipids. This suggested that other air pollutants may alter the associations between PM_2.5_ and triglyceride, which requires further research.

In our study, PM_2.5_ was positively associated with the low-density lipoprotein cholesterol concentration. Most previous studies investigated long-term, rather than short-term, exposure effects of PM_2.5_ to low-density lipoprotein cholesterol concentration^23–25^. Our study provides new evidence for the effect of short-term exposure. Long-term PM_2.5_ exposure was shown to be negatively associated with high-density lipoprotein cholesterol concentration^18^, inconsistent with our findings, indicating that differential exposure durations (short- versus long-term) may also have an impact on the results.

Previous studies have been heterogenous concerning the effect of PM_2.5_ on total cholesterol concentration. A study in Shanghai showed no significant association between total cholesterol and PM_2.5_^26^. However, another study is consistent with our results^27^, showing PM_2.5_ exposure was associated with an elevated total cholesterol concentration. The difference of exposure durations may explain the inconsistency because we evaluated the short-term effect of PM_2.5_, while the study in shanghai explored the long-term exposure. A study among college students^20^ showed that short-term PM_2.5_ exposure was negatively associated with total cholesterol concentration, the inconsistency may be attributed to the difference of sample size and age. Recent research showed that long- term PM_2.5_ exposure was negatively correlated with the risk of hyperlipidemia^28^, however, in our study, the OR of every 10 μg/m^3^ increase in PM_2.5_ for hyperlipidemia population was 1.009, which suggested that short-term PM_2.5_ exposure was a risk factor for hyperlipidemia. Different life-styles and areas may explain the inconsistency. In our study, the effect of short-term PM_2.5_ exposure on the low-density lipoprotein cholesterol concentration and total cholesterol concentration could be modified by age and the older were more susceptible to PM_2.5_ exposure, which may be due to hypometabolism and/or hypoimmunity. Previous studies support these findings^29–31^.

Our study has several limitations. First, our study was a time-series study, limiting our ability to account for reverse causation or time-specific confounding. Second, the fixed environmental monitoring station was used to estimate personal PM_2.5_ exposure, which cannot be equated entirely with individual exposure. Lastly, although our dataset was large and community-based, we did not have available several other characteristics which may be associated with PM_2.5_ exposure and blood lipid-related indicators, such as exercise, smoking, and medical history. Therefore, unmeasured and residual confounding is possible.

## Conclusions

PM_2.5_ was positively correlated with low-density lipoprotein cholesterol and total cholesterol, and negatively correlated with triglyceride, indicating PM_2.5_ can potentially affect health through blood lipid levels.

## Supplementary Information

Supplementary Tables.

## Data Availability

The datasets used and/or analyzed during the current study are available from the corresponding author on reasonable request.

## Abbreviations

SD: Standard deviation
IQR: Interquartile range
95%CI: 95% Confidence interval
OR: Odds ratio
PM_2.5_: Particulate matter with an aerodynamic matter smaller than 2.5 μm
PM_10_: Particulate matter with an aerodynamic matter smaller than 10 μm
NO_2_: Nitrogen dioxide
SO_2_: Sulphur dioxide
O_3_: Ozone
CO: Carbon monoxide
GAM: Generalized additive model
AIC: Akaike information criterion
