# Supplementary material for: Effects of short-term PM2.5 exposure on blood lipids among 197,957 people in eastern China
Source: Sci Rep. 2023 Mar 18;13:4505. doi: 10.1038/s41598-023-31513-y (PMC10024762; doi:10.1038/s41598-023-31513-y)
Supplement: Supplementary file 1 — Supplementary Tables. [file 41598_2023_31513_MOESM1_ESM.docx]

Supplementary Table 1. Spearman rank correlation coefficients between daily average meteorological factors and air pollutant concentrations in Yixing city during the study period.

| **Variables** | **Temperature** | **Atmospheric pressure** | **Wind speed** | **Relative humidity** | **PM_2.5_** | **PM_10_** | **SO_2_** | **NO_2_** | **O_3_** | **CO** |
| --- | --- | --- | --- | --- | --- | --- | --- | --- | --- | --- |
| Temperature | 1.00 | -0.90^**^ | 0.10^**^ | 0.00 | -0.40^**^ | -0.38^**^ | -0.29^**^ | -0.50^**^ | 0.62^**^ | -0.46^**^ |
| Atmospheric pressure |  | 1.00 | - 0.13^**^ | -0.15^**^ | 0.32^**^ | 0.35^**^ | 0.31^**^ | 0.47^**^ | -0.53^**^ | 0.35^**^ |
| Wind speed |  |  | 1.00 | -0.07^**^ | -0.34^**^ | -0.30^**^ | -0.14^**^ | -0.45^**^ | 0.04 | -0.33^**^ |
| Relative humidity |  |  |  | 1.00 | -0.04 | -0.28^**^ | -0.27^**^ | -0.04 | -0.44^**^ | 0.13^**^ |
| PM_2.5_ |  |  |  |  | 1.00 | 0.92^**^ | 0.62^**^ | 0.67^**^ | -0.03 | 0.76^**^ |
| PM_10_ |  |  |  |  |  | 1.00 | 0.66^**^ | 0.70^**^ | 0.04^*^ | 0.67^**^ |
| SO_2_ |  |  |  |  |  |  | 1.00 | 0.51^**^ | -0.02 | 0.53^**^ |
| NO_2_ |  |  |  |  |  |  |  | 1.00 | -0.27^**^ | 0.61^**^ |
| O_3_ |  |  |  |  |  |  |  |  | 1.00 | -0.23^**^ |
| CO |  |  |  |  |  |  |  |  |  | 1.00 |

^**^: *P* <0.01; ^*^: *P* <0.05.

Supplementary Table 2. The df of meteorological factors in the analyze of effects of PM_2.5_ on blood lipids.

| **Indicators** | **the df of meteorological factors** | | |
| --- | --- | --- | --- |
|  | **Temperature** | **Relative humidity** | **Wind speed** |
| Triglyceride | 10 | 10 | 7 |
| Low-density lipoprotein cholesterol | 8 | 9 | 10 |
| High-density lipoprotein cholesterol | 10 | 8 | 9 |
| Total cholesterol | 10 | 9 | 6 |

Supplementary Table 3. Effect modification of sex and age on the associations between PM_2.5_ and blood lipid levels^a^.

| **Indicators** | **Complete study population** | | **Persons with normal blood lipid levels^b^** | |
| --- | --- | --- | --- | --- |
|  | **Sex** | **Age** | **Sex** | **Age** |
| Triglyceride | 1.16 | 1.80 | 1.58 | 2.11 |
| Low-density lipoprotein cholesterol | 0.45 | 2.25 | 0.59 | 0.57 |
| High-density lipoprotein cholesterol | 0.34 | 1.86 | 1.74 | 1.36 |
| Total cholesterol | 1.57 | 1.99 | 0.95 | 0.66 |

^a^: A value larger than 1.96 indicated that the association between PM_2.5_ and the blood lipids were modified by sex or age.

^b：^Persons with normal blood lipid levels were people with total cholesterol <6.2 mmol/L, triglyceride <2.3 mmol/L, low-density lipoprotein cholesterol <4.1 mmol/L and high-density lipoprotein cholesterol >1.0 mmol/L.

Supplementary Table 4. The effect of every 10 μg/m^3^ increase in PM_2.5_ for hyperlipidemia population.

| **Groups** | **N (%)** | **OR** | **95% CI** |
| --- | --- | --- | --- |
| Total | 195,927 | 1.003^*^ | (1.001, 1.004) |
| Male | 109,166 (55.72) | 1.004^*^ | (1.002, 1.007) |
| Female | 86,761 (44.28) | 1.001 | (0.999, 1.004) |
| Age <60 years | 154,330 (78.77) | 1.003^*^ | (1.001, 1.005) |
| Age ≥60 years | 41,597 (21.23) | 1.002 | (0.998, 1.006) |

^*^: *P* <0.05

Supplementary Table 5. The effect of every 10 μg/m^3^ increase in PM_2.5_ for persons with normal blood lipid levels^a^.

| **Indicators** | **OR** | **95% CI** |
| --- | --- | --- |
| Triglyceride | 0.998 | (0.996, 0.999) |
| Low-density lipoprotein cholesterol | 1.001 | (1.000, 1.001) |
| High-density lipoprotein cholesterol | 1.002 | (1.001, 1.003) |
| Total cholesterol | 1.003 | (1.001, 1.004) |

^a^: We applied lag 0-6 days for triglyceride, lag 0-3 days for low-density lipoprotein cholesterol, lag 0-5 days for high-density lipoprotein cholesterol, and lag 0-7 days for total cholesterol.

Supplementary Table 6. Estimated changes in the blood lipids for every 10 μg/m^3^ increase in SO_2_.

| **Indicators** | **Complete study population** | | **Persons with normal blood lipid levels^a^** | |
| --- | --- | --- | --- | --- |
|  | **Single-pollutant model** | **Multi-pollutant model^b^** | **Single-pollutant model** | **Multi-pollutant model^b^** |
| Triglyceride (%) | 0.7824 (-0.0526, 1.6245) | 1.7837 (0.8617, 2.7142) | -0.5457 (-1.2520, 0.1656) | -0.1216 (-0.8913, 0.6540) |
| Low-density lipoprotein cholesterol (mmol/ L) | 0.0071(-0.0025, 0.0166) | 0.0079 (-0.0025, 0.0184) | 0.0097 (0.0003, 0.0191) | 0.0083 (-0.0020, 0.0186) |
| High-density lipoprotein cholesterol (mmol/L) | -0.0209 (-0.0250, -0.0169) | -0.0036 (-0.0380, -0.0292) | -0.0126 (-0.0170, -0.0082) | -0.0217 (-0.0265, -0.0168) |
| Total cholesterol (mmol/L) | 0.0252 (0.0115, 0.0390) | 0.0415 (0.0264, 0.0565) | 0.0170 (0.0042, 0.0297) | 0.0299 (0.0160, 0.0438) |

^a^: Persons with normal blood lipid levels were people with total cholesterol <6.2mmol/L, triglyceride <2.3 mmol/L, low-density lipoprotein cholesterol <4.1 mmol/L and high-density lipoprotein cholesterol >1.0 mmol/L. We applied lag 0-6 days for triglyceride, lag 0-6 days for low-density lipoprotein cholesterol, lag 0-5 days for high-density lipoprotein cholesterol, and lag 0-7 days for total cholesterol.

^b^: Adjusted for CO, NO_2_ and O_3_.

Supplementary Table 7. Estimated changes in the blood lipids for every 10 μg/m^3^ increase in SO_2_ at different lag days^a^.

| **Indicators and lag days** | **Entire population** | **Persons with normal blood lipid levels^b^** |
| --- | --- | --- |
|  |  |  |
| Triglyceride (%) |  |  |
| 0-1 days | -0.2413 (-0.9041,0.4259) | -0.7027 (-1.2655, -0.1376) |
| 0-2 days | -0.3045 (-1.0119, 0.4079) | -0.9250 (-1.5253, -0.3210) |
| 0-3 days | 0.1073 (-0.6473, 0.8675) | -0.8674 (-1.5075, -0.2232) |
| 0-4 days | 0.4071 (-0.3807, 1.2102) | -0.7646 (-1.4320, -0.0926) |
| 0-5 days | 0.7862 (-0.0291, 1.6081) | -0.5714 (-1.2614, 0.1235) |
| 0-6 days | 0.7824 (-0.0526, 1.6245) | -0.5457 (-1.2520, 0.1656) |
| 0-7 days | 1.0646 (0.2162, 1.9202) | -0.4001 (-1.1196, 0.3246) |
| Low-density lipoprotein cholesterol (mmol/L) |  |  |
| 0-1 days | 0.0026 (-0.0050, 0.0102) | 0.0050 (-0.0025, 0.0125) |
| 0-2 days | 0.0025 (-0.0057, 0.0106) | 0.0024 (-0.0056, 0.0104) |
| 0-3 days | 0.0080 (-0.0006, 0.0166) | 0.0065 (-0.0020, 0.0150) |
| 0-4 days | 0.0083 (-0.0007, 0.0173) | 0.0085 (-0.0003, 0.0174) |
| 0-5 days | 0.0065 (-0.0028, 0.0158) | 0.0081 (-0.0077, 0.0238) |
| 0-6 days | 0.007 1(-0.0025, 0.0166) | 0.0097 (0.0003, 0.0191) |
| 0-7 days | 0.0115 (0.0018, 0.0211) | 0.0130 (0.0034, 0.0225) |
| High-density lipoprotein cholesterol (mmol/L) |  |  |
| 0-1 days | -0.0166 (-0.0199, -0.0132) | -0.0104 (-0.0140, -0.0068) |
| 0-2 days | -0.0167 (-0.0202, -0.0131) | -0.0101 (-0.0140, -0.0062) |
| 0-3 days | -0.0182 (-0.0220, -0.0145) | -0.0109 (-0.0150, -0.0068) |
| 0-4 days | -0.0191 (-0.0230, -0.0152) | -0.0115 (-0.0157, -0.0072) |
| 0-5 days | -0.0209 (-0.0250, -0.0169) | -0.0126 (-0.0170, -0.0082) |
| 0-6 days | -0.0234 (-0.0276, -0.0193) | -0.0146 (-0.0191, -0.0101) |
| 0-7 days | -0.0226 (-0.0268, -0.0184) | -0.0133 (-0.0180, -0.0087) |
| Total cholesterol (mmol/L) |  |  |
| 0-1 days | 0.0036 (-0.0072 ,0.0144) | 0.0054 (-0.0045, 0.0154) |
| 0-2 days | 0.0090 (-0.0026 ,0.0206) | 0.0072 (-0.0034, 0.0179) |
| 0-3 days | 0.0143 (0.0021, 0.0265) | 0.0084 (-0.0029, 0.0197) |
| 0-4 days | 0.0144 (0.0017 ,0.0272) | 0.0098 (-0.0019, 0.0215) |
| 0-5 days | 0.0166 (0.0035, 0.0298) | 0.0095 (-0.0025, 0.0215) |
| 0-6 days | 0.0185 (0.0050, 0.0321) | 0.0135 (0.0010, 0.0261) |
| 0-7 days | 0.0252 (0.0115, 0.0390) | 0.0170 (0.0042, 0.0297) |
|  |  |  |

^a^: Adjusted for time, day of the week, sex, age, temperature, wind speed and relative humidity.

^b^: Persons with normal blood lipid levels were people with total cholesterol <6.2 mmol/L, triglyceride <2.3 mmol/L, low-density lipoprotein cholesterol <4.1 mmol/L and high-density lipoprotein cholesterol >1.0 mmol/L

Supplementary Table 8. Estimated changes in the blood lipids for every 10 μg/m^3^ increase in NO_2_.

| **Indicators** | **Complete study population^a^** | | **Persons with normal blood lipid levels^a^** | |
| --- | --- | --- | --- | --- |
|  | **Single-pollutant model** | **Multi-pollutant model^b^** | **Single-pollutant model** | **Multi-pollutant model^b^** |
| Triglyceride (%) | -0.7023 (-1.1189, -0.2840) | -1.1909 (-1.6522, -0.7232) | -0.4601 (-0.8109, -0.1081) | -0.5974 (-0.9882, -0.2050) |
| Low-density lipoprotein cholesterol (mmol/ L) | -0.0026 (-0.0074, 0.0023) | -0.0048 (-0.0102, 0.0006) | 0.0024 (-0.0023, 0.0071) | 0.0004 (-0.0049, 0.0056) |
| High-density lipoprotein cholesterol (mmol/L) | 0.0020 (0.0000, 0.0040) | 0.0021 (-0.0002, 0.0043) | 0.0019 (-0.0002, 0.0041) | 0.0014 (-0.0010, 0.0038) |
| Total cholesterol (mmol/L) | -0.0261 (-0.0332, -0.0190) | -0.0427 (-0.0506, -0.0348) | -0.0193 (-0.0257, -0.0128) | -0.0302 (-0.0374, -0.0230) |

^a^: Persons with normal blood lipid levels were people with total cholesterol <6.2mmol/L, triglyceride <2.3 mmol/L, low-density lipoprotein cholesterol <4.1 mmol/L and high-density lipoprotein cholesterol >1.0 mmol/L. We applied lag 0-6 days for triglyceride, lag 0-6 days for low-density lipoprotein cholesterol, lag 0-5 days for high-density lipoprotein cholesterol, and lag 0-7 days for total cholesterol.

^b^: Adjusted for CO, SO_2_ and O_3_.

Supplementary Table 9. Estimated changes in the blood lipids for every 10 μg/m^3^ increase in NO_2_ at different lag days^a^.

| **Indicators and lag days** | **Entire population** | **Persons with normal blood lipid levels^b^** |
| --- | --- | --- |
|  |  |  |
| Triglyceride (%) |  |  |
| 0-1 days | -0.2887 (-0.5711, -0.0055) | -0.0351 (-0.2746, 0.2051) |
| 0-2 days | -0.3482 (-0.6229, -0.0325) | -0.1270 (-0.3941, 0.1407) |
| 0-3 days | -0.5835 (-0.9305, -0.2353) | -0.3593 (-0.6537, -0.0639) |
| 0-4 days | -0.6067 (-0.9777, -0.2343) | -0.4108 (-0.7255, -0.0950) |
| 0-5 days | -0.4278 (-0.8220, -0.0320) | -0.2906 (-0.6246, 0.0446) |
| 0-6 days | -0.7023 (-1.1189, -0.2840) | -0.4601 (-0.8109, -0.1081) |
| 0-7 days | -0.6602 (-1.0900, -0.2886) | -0.4788 (-0.8439, -0.1224) |
| Low-density lipoprotein cholesterol (mmol/L) |  |  |
| 0-1 days | 0.0034 (-0.0033, 0.0101) | 0.0042 (0.0011, 0.0074) |
| 0-2 days | -0.0020 (-0.0060, 0.0020) | 0.0015 (-0.0021, 0.0050) |
| 0-3 days | -0.0038 (-0.0081, 0.0005) | 0.0002 (-0.0037, 0.0041) |
| 0-4 days | -0.0036 (-0.0081, 0.0010) | 0.0001 (-0.0041, 0.0043) |
| 0-5 days | -0.0036 (-0.0081, 0.0010) | 0.0018 (-0.0026, 0.0063) |
| 0-6 days | -0.0026 (-0.0074, 0.0023) | 0.0024 (-0.0023, 0.0071) |
| 0-7 days | 0.0022 (-0.0028, 0.0072) | 0.0061 (0.0012, 0.0110) |
| High-density lipoprotein cholesterol (mmol/L) |  |  |
| 0-1 days | 0.0027 (0.0012, 0.0041) | 0.0025 (0.0010, 0.0041) |
| 0-2 days | 0.0032 (0.0016, 0.0048) | 0.0032 (0.0015, 0.0049) |
| 0-3 days | 0.0037 (0.0019, 0.0054) | 0.0035 (0.0016, 0.0054) |
| 0-4 days | 0.0033 (0.0014, 0.0051) | 0.0030 (0.0009, 0.0050) |
| 0-5 days | 0.0020 (0.0000, 0.0040) | 0.0019 (-0.0002, 0.0041) |
| 0-6 days | 0.0015 (-0.0006, 0.0036) | 0.0016 (-0.0007, 0.0039) |
| 0-7 days | 0.0015 (-0.0007, 0.0037) | 0.0020 (-0.0003, 0.0044) |
| Total cholesterol (mmol/L) |  |  |
| 0-1 days | -0.0054 (-0.0099, -0.0010) | -0.0042 (-0.0082, -0.0001) |
| 0-2 days | -0.0086 (-0.0137, -0.0034) | -0.0073 (-0.0018, -0.0028) |
| 0-3 days | -0.0154 (-0.0211 ,-0.0098) | -0.0120 (-0.0171, -0.0069) |
| 0-4 days | -0.0223 (-0.0284, -0.0163) | -0.0165 (-0.0220, -0.0110) |
| 0-5 days | -0.0262 (-0.0326, -0.0198) | -0.0181 (-0.0239, -0.0122) |
| 0-6 days | -0.0290 (-0.0358, -0.0221) | -0.0206 (-0.0268, -0.0144) |
| 0-7 days | -0.0261 (-0.0322, -0.0190) | -0.0193 (-0.0257, -0.0128) |
|  |  |  |

^a^: Adjusted for time, day of the week, sex, age, temperature, wind speed and relative humidity.

^b^: Persons with normal blood lipid levels were people with total cholesterol <6.2 mmol/L, triglyceride <2.3 mmol/L, low-density lipoprotein cholesterol <4.1 mmol/L and high-density lipoprotein cholesterol >1.0 mmol/L

Supplementary Table 10. Estimated changes in the blood lipids for every 10 μg/m^3^ increase in O_3_.

| **Indicators** | **Complete study population^a^** | | **Persons with normal blood lipid levels^a^** | |
| --- | --- | --- | --- | --- |
|  | **Single-pollutant model** | **Multi-pollutant model^b^** | **Single-pollutant model** | **Multi-pollutant model^b^** |
| Triglyceride (%) | -0.7207 (-0.8412, -0.6001) | -0.8585 (-0.9906 ,-0.7262) | -0.6042 (-0.70-73, -0.5009) | -0.6964 (-0.8090, -0.5836) |
| Low-density lipoprotein cholesterol (mmol/ L) | 0.0036 (0.0022, 0.0049) | 0.0036 (0.0022, 0.0051) | 0.0012 (-0.0001, 0.0025) | 0.0012 (-0.0003, 0.0026) |
| High-density lipoprotein cholesterol (mmol/L) | 0.0025 (0.0019, 0.0031) | 0.0010 (0.0004, 0.0017) | 0.0011 (0.0005, 0.0018) | -0.0001 (-0.0008, 0.0006) |
| Total cholesterol (mmol/L) | 0.0031 (0.0011, 0.0052) | 0.0002 (-0.0020, 0.0025) | 0.0008 (-0.0011, 0.0027) | -0.0011 (-0.0032, 0.0009) |

^a^: Persons with normal blood lipid levels were people with total cholesterol <6.2mmol/L, triglyceride <2.3 mmol/L, low-density lipoprotein cholesterol <4.1 mmol/L and high-density lipoprotein cholesterol >1.0 mmol/L. We applied lag 0-6 days for triglyceride, lag 0-6 days for low-density lipoprotein cholesterol, lag 0-5 days for high-density lipoprotein cholesterol, and lag 0-7 days for total cholesterol.

^b^: Adjusted for CO, NO_2_ and SO_2_.

Supplementary Table 11. Estimated changes in the blood lipids for every 10 μg/m^3^ increase in O_3_ at different lag days^a^.

| **Indicators and lag days** | **Entire population** | **Persons with normal blood lipid levels^b^** |
| --- | --- | --- |
|  |  |  |
| Triglyceride (%) |  |  |
| 0-1 days | -0.4066 (-0.4913, -0.3217) | -0.2932 (-0.3651, -0.2211) |
| 0-2 days | -0.4881 (-0.5808, -0.3954) | -0.3607 (-0.4393, -0.2822) |
| 0-3 days | -0.5294 (-0.6308, -0.4279) | -0.4119 (-0.4979, -0.3259) |
| 0-4 days | -0.6159 (-0.7246, -0.5070) | -0.4923 (-0.5848, -0.3998) |
| 0-5 days | -0.6906 (-0.8064, -0.5747) | -0.5590 (-0.6583, -0.4597) |
| 0-6 days | -0.7207 (-0.8412 ,-0.6001) | -0.6042 (-0.7073, -0.5009) |
| 0-7 days | -0.7786 (-0.9021, -0.6548) | -0.6411 (-0.7467, -0.5355) |
| Low-density lipoprotein cholesterol (mmol/L) |  |  |
| 0-1 days | 0.0030 (0.0020, 0.0040) | 0.0008 (-0.0001, 0.0018) |
| 0-2 days | 0.0030 (0.0020, 0.0041) | 0.0008( -0.0003, 0.0018) |
| 0-3 days | 0.0028 (0.0017, 0.0040) | 0.0005 (-0.0006, 0.0016) |
| 0-4 days | 0.0038 (0.0026, 0.0051) | 0.0013 (0.0001, 0.0025) |
| 0-5 days | 0.0036 (0.0022, 0.0049) | 0.0012 (-0.0001, 0.0025) |
| 0-6 days | 0.0030 (0.0016, 0.0044) | 0.0007 (-0.0007, 0.0021) |
| 0-7 days | 0.0027 (0.0012, 0.0041) | 0.0005 (-0.0009, 0.0020) |
| High-density lipoprotein cholesterol (mmol/L) |  |  |
| 0-1 days | 0.0019 (0.0014, 0.0023) | 0.0009 (0.0004, 0.0013) |
| 0-2 days | 0.0021 (0.0017, 0.0026) | 0.0010 (0.0005, 0.0015) |
| 0-3 days | 0.0022 (0.0017, 0.0027) | 0.0010 (0.0005, 0.0016) |
| 0-4 days | 0.0028 (0.0022, 0.0033) | 0.0014 (0.0008, 0.0020) |
| 0-5 days | 0.0025 (0.0019, 0.0031) | 0.0011 (0.0005, 0.0018) |
| 0-6 days | 0.0028 (0.0022, 0.0034) | 0.0013 (0.0006, 0.0019) |
| 0-7 days | 0.0029 (0.0023, 0.0036) | 0.0014 (0.0007, 0.0020) |
| Total cholesterol (mmol/L) |  |  |
| 0-1 days | 0.0035 (0.0021, 0.0049) | 0.0013 (0.0000, 0.0025) |
| 0-2 days | 0.0034 (0.0019, 0.0049) | 0.0010 (-0.0004, 0.0024) |
| 0-3 days | 0.0034 (0.0017, 0.0050) | 0.0009 (-0.0007, 0.0024) |
| 0-4 days | 0.0037 (0.0019, 0.0054) | 0.0009 (-0.0007, 0.0025) |
| 0-5 days | 0.0033 (0.0014, 0.0052) | 0.0012 (-0.0005, 0.0029) |
| 0-6 days | 0.0034 (0.0014, 0.0054) | 0.0012 (-0.0006, 0.0030) |
| 0-7 days | 0.0031 (0.0011, 0.0052) | 0.0008 (-0.0011, 0.0027) |
|  |  |  |

^a^: Adjusted for time, day of the week, sex, age, temperature, wind speed and relative humidity.

^b^: Persons with normal blood lipid levels were people with total cholesterol <6.2 mmol/L, triglyceride <2.3 mmol/L, low-density lipoprotein cholesterol <4.1 mmol/L and high-density lipoprotein cholesterol >1.0 mmol/L

Supplementary Table 12. Estimated changes in the blood lipids for every 10 μg/m^3^ increase in PM_10_.

| **Indicators** | **Complete study population^a^** | | **Persons with normal blood lipid levels^a^** | |
| --- | --- | --- | --- | --- |
|  | **Single-pollutant model** | **Multi-pollutant model^b^** | **Single-pollutant model** | **Multi-pollutant model^b^** |
| Triglyceride (%) | -0.342 (-0.4831 ,-0.1851) | -0.3475 (-0.5269, -0.1677) | -0.2613 (-0.3873, -0.1351) | -0.1756 (-0.3269, -0.0242) |
| Low-density lipoprotein cholesterol (mmol/ L) | 0.0062 (0.0044, 0.0080) | 0.0063 (0.0042 ,0.0085) | 0.0049 (0.0032, 0.0066) | 0.0046 (0.0024, 0.0067) |
| High-density lipoprotein cholesterol (mmol/L) | 0.0002 (-0.0005, 0.0009) | -0.0020 (-0.0028, -0.0011) | -0.0002 (-0.0010, 0.0006) | -0.0019 (-0.0028, -0.0010) |
| Total cholesterol (mmol/L) | 0.001 6(-0.0010, 0.0041) | -0.0036 (-0.0066, -0.0005) | 0.0002 (0.0024, 0.0067) | -0.0029 (-0.0058, -0.0001) |

^a^: Persons with normal blood lipid levels were people with total cholesterol <6.2mmol/L, triglyceride <2.3 mmol/L, low-density lipoprotein cholesterol <4.1 mmol/L and high-density lipoprotein cholesterol >1.0 mmol/L. We applied lag 0-6 days for triglyceride, lag 0-6 days for low-density lipoprotein cholesterol, lag 0-5 days for high-density lipoprotein cholesterol, and lag 0-7 days for total cholesterol.

^b^: Adjusted for CO, SO_2_ and O_3_.

Supplementary Table 13. Estimated changes in the blood lipids for every 10 μg/m^3^ increase in PM_10_ at different lag days^a^.

| **Indicators and lag days** | **Entire population** | **Persons with normal blood lipid levels^b^** |
| --- | --- | --- |
|  |  |  |
| Triglyceride (%) |  |  |
| 0-1 days | -0.1939 (-0.2916 ,-0.0961) | -0.1231 (-0.2060, -0.0402) |
| 0-2 days | -0.2687 (-0.3787, -0.1587) | -0.2201 (-0.3135, -0.1266) |
| 0-3 days | -0.2843 (-0.4054, -0.1630) | -0.2762 (-0.3795, -0.1728) |
| 0-4 days | -0.2994 (-0.4306, -0.1679) | -0.3000 (-0.4123, -0.1877) |
| 0-5 days | -0.2349 (-0.3761, -0.0935) | -0.2206 (-0.3415, -0.0995) |
| 0-6 days | -0.3342 (-0.4831, -0.1851) | -0.2613 (-0.3873, -0.1351) |
| 0-7 days | -0.4144 (-0.5684, -0.2602) | -0.3569 (-0.4893, -0.2242) |
| Low-density lipoprotein cholesterol (mmol/L) |  |  |
| 0-1 days | 0.0024 (0.0013, 0.0035) | 0.0006 (-0.0005, 0.0017) |
| 0-2 days | 0.0025 (0.0012, 0.0037) | 0.0004 (-0.0008, 0.0016) |
| 0-3 days | 0.0030 (0.0016, 0.0044) | 0.0010 (-0.0003, 0.0024) |
| 0-4 days | 0.0035 (0.0020, 0.0050) | 0.0018 (0.0003, 0.0033) |
| 0-5 days | 0.0038 (0.0022, 0.0054) | 0.0026 (0.0010, 0.0042) |
| 0-6 days | 0.0049 (0.0032, 0.0066) | 0.0035 (0.0018, 0.0052) |
| 0-7 days | 0.0062 (0.0044, 0.0080) | 0.0049 (0.0032, 0.0066) |
| High-density lipoprotein cholesterol (mmol/L) |  |  |
| 0-1 days | 0.0007 (0.0002, 0.0012) | 0.0004 (-0.0001, 0.0010) |
| 0-2 days | 0.0007 (0.0012, 0.0013) | 0.0004 (-0.0002, 0.0010) |
| 0-3 days | 0.0006 (0.0000, 0.0012) | 0.0003 (-0.0004, 0.0010) |
| 0-4 days | 0.0007 (0.0001, 0.0014) | 0.0004 (-0.0003, 0.0011) |
| 0-5 days | 0.0002 (-0.0005, 0.0009) | -0.0002 (-0.0010, 0.0006) |
| 0-6 days | 0.0002 (-0.0005, 0.0010) | -0.0003 (-0.0012, 0.0005) |
| 0-7 days | 0.0007 (-0.0001, 0.0015) | -0.0001 (-0.0009, 0.0008) |
| Total cholesterol (mmol/L) |  |  |
| 0-1 days | 0.0019 (0.0004 ,0.0035) | 0.0004 (-0.0010, 0.0018) |
| 0-2 days | 0.0020 (0.0002, 0.0038) | 0.0002 (-0.0014, 0.0017) |
| 0-3 days | 0.0017 (-0.0003, 0.0037) | 0.0001 (-0.0017, 0.0019) |
| 0-4 days | 0.0016 (-0.0006, 0.0037) | 0.0004 (-0.0015, 0.0022) |
| 0-5 days | 0.0011 (-0.0013, 0.0035) | 0.0008 (-0.0012, 0.0029) |
| 0-6 days | 0.0016 (-0.0010, -0.0041) | 0.0003 (-0.0020, 0.0025) |
| 0-7 days | 0.0006 (-0.0005, 0.0017) | 0.0002 (-0.0022, 0.0025) |
|  |  |  |

^a^: Adjusted for time, day of the week, sex, age, temperature, wind speed and relative humidity.

^b^: Persons with normal blood lipid levels were people with total cholesterol <6.2 mmol/L, triglyceride <2.3 mmol/L, low-density lipoprotein cholesterol <4.1 mmol/L and high-density lipoprotein cholesterol >1.0 mmol/L

Supplementary Table 14. Estimated changes in the blood lipids for every 1 mg/m^3^ increase in CO.

| **Indicators** | **Complete study population^a^** | | **Persons with normal blood lipid levels^a^** | |
| --- | --- | --- | --- | --- |
|  | **Single-pollutant model** | **Multi-pollutant model^b^** | **Single-pollutant model** | **Multi-pollutant model^b^** |
| Triglyceride (%) | -0.8414 (-2.2949, 0.6337) | 3.9157 (2.1259, 5.7369) | -0.9841 (-0.2182, 0.2656) | 3.2487 (1.7348, 4.7850) |
| Low-density lipoprotein cholesterol (mmol/ L) | 0.0279 (0.0147, 0.0411) | 0.0125 (-0.0030, 0.0280) | 0.0158 (0.0030, 0.0287) | 0.0076 (-0.0075, 0.0226) |
| High-density lipoprotein cholesterol (mmol/L) | 0.0520 (0.0448, 0.0591) | 0.0618 (0.0533, 0.0702) | 0.0376 (0.0299, 0.0454) | 0.0471 (0.0379, 0.0562) |
| Total cholesterol (mmol/L) | 0.0597 (0.0352, 0.0842) | 0.0878 (0.0588, 0.1167) | 0.0337 (0.0112, 0.0562) | 0.0600 (0.0334, 0.0856) |

^a^: Persons with normal blood lipid levels were people with total cholesterol <6.2mmol/L, triglyceride <2.3 mmol/L, low-density lipoprotein cholesterol <4.1 mmol/L and high-density lipoprotein cholesterol >1.0 mmol/L. We applied lag 0-6 days for triglyceride, lag 0-6 days for low-density lipoprotein cholesterol, lag 0-5 days for high-density lipoprotein cholesterol, and lag 0-7 days for total cholesterol.

^b^: Adjusted for SO_2_, NO_2_ and O_3_.

Supplementary Table 15. Estimated changes in the blood lipids for every 1 mg/m^3^ increase in CO at different lag days^a^.

| **Indicators and lag days** | **Entire population** | **Persons with normal blood lipid levels^b^** |
| --- | --- | --- |
| Triglyceride (%) |  |  |
| 0-1 days | -1.2995 (-2.4402, -0.1454) | -1.1750 (-2.1419, -0.1987) |
| 0-2 days | -1.5597 (-2.7859, -0.3181) | -1.5809 (-2.6205, -0.5302) |
| 0-3 days | -1.4070 (-2.6946, -0.1024) | -1.7397 (-2.8308, -.06364) |
| 0-4 days | -1.6315 (-2.9733, -0.2713) | -2.1417 (-3.2790, -0.9911) |
| 0-5 days | -0.9346 (-2.3442, 0.4954) | -1.4691 (-2.6972, -0.2255) |
| 0-6 days | -0.8414 (-2.2949, 0.6337) | -0.9841 (-2.2182, 0.2656) |
| 0-7 days | -0.8173 (-2.2968, 0.6846) | -0.7984 (-2.0594, 0.4789) |
| Low-density lipoprotein cholesterol (mmol/L) |  |  |
| 0-1 days | 0.0279 (0.0147, 0.0411) | 0.0158 (0.0030, 0.0287) |
| 0-2 days | 0.0225 (0.0083, 0.0411) | 0.0108 (-0.0031, 0.0247) |
| 0-3 days | 0.0230 (0.0080, 0.0379) | 0.0135 (-0.0012, 0.0281) |
| 0-4 days | 0.020 1(0.0044, 0.0357) | 0.0144 (-0.0009, 0.0297) |
| 0-5 days | 0.0143 (-0.0020, 0.0305) | 0.0118 (-0.0041, 0.0278) |
| 0-6 days | 0.0184 (0.0016, 0.0352) | 0.0166 (0.0002, 0.0331) |
| 0-7 days | 0.0261 (0.0089, 0.0432) | 0.0251 (0.0083, 0.0419) |
| High-density lipoprotein cholesterol (mmol/L) |  |  |
| 0-1 days | 0.0391 (0.0333, 0.0449) | 0.0289 (0.0227, 0.0352) |
| 0-2 days | 0.0444 (0.0381, 0.0506) | 0.0308 (0.0241, 0.0376) |
| 0-3 days | 0.0461 (0.0395, 0.0526) | 0.0322 (0.0251, 0.0393) |
| 0-4 days | 0.0519 (0.0450, 0.0588) | 0.0372 (0.0297, 0.0446) |
| 0-5 days | 0.0520 (0.0448, 0.0591) | 0.0376 (0.0299, 0.0454) |
| 0-6 days | 0.0536 (0.0462, 0.0609) | 0.0362 (0.0282, 0.0441) |
| 0-7 days | 0.0583 (0.0508, 0.0659) | 0.0382 (0.0301, 0.0464) |
| Total cholesterol (mmol/L) |  |  |
| 0-1 days | 0.0577 (0.0391, 0.0763) | 0.0331 (0.0162, 0.0500) |
| 0-2 days | 0.0637 (0.0434, 0.0840) | 0.0350 (0.0167, 0.0533) |
| 0-3 days | 0.0627 (0.0414, 0.0840) | 0.0350 (0.0155, 0.0545) |
| 0-4 days | 0.0568 (0.0346, 0.0790) | 0.0319 (0.0118, 0.0520) |
| 0-5 days | 0.0570 (0.0338, 0.0801) | 0.0342 (0.0132, 0.0552) |
| 0-6 days | 0.0579 (0.0339, 0.0819) | 0.0353 (0.0133, 0.0573) |
| 0-7 days | 0.0597 (0.0352, 0.0842) | 0.0337 (0.0112, 0.0562) |
|  |  |  |

^a^: Adjusted for time, day of the week, sex, age, temperature, wind speed and relative humidity.

^b^: Persons with normal blood lipid levels were people with total cholesterol <6.2 mmol/L, triglyceride <2.3 mmol/L, low-density lipoprotein cholesterol <4.1 mmol/L and high-density lipoprotein cholesterol >1.0 mmol/L
